# The Genetics of Early-Stage Melanoma in a Veteran Population

**DOI:** 10.3389/fonc.2022.887768

**Published:** 2022-05-30

**Authors:** Kevin Cheung, Aaron D. Bossler, Sarah L. Mott, Megan Zeisler, Julie McKillip, Yousef Zakharia, Brian L. Swick, Jennifer G. Powers

**Affiliations:** ^1^ Department of Dermatology, University of Iowa, Iowa City, IA, United States; ^2^ Department of Pathology, H. Lee Moffitt Cancer Center, Tampa, FL, United States; ^3^ Holden Comprehensive Cancer Center, University of Iowa, Iowa City, IA, United States

**Keywords:** melanoma, military personnel, veterans, genetic predisposition, risk factors, proto-oncogene, BRAF, TP53

## Abstract

To improve understanding of the genetic signature of early-stage melanomas in Veterans, hotspot mutation profiling using next-generation sequencing (NGS) was performed on melanoma tissue samples from patients at the Iowa City Veterans Affairs Medical Center (VAMC). Genetic analysis identified BRAF (36.3%), TP53 (25.9%), NRAS (19.3%), CDKN2A (11.1%), KIT (8.1%), and BAP1 (7.4%) mutations with the highest prevalence. Although common variants in BRAF were detected at lower rates than what is reported for the general population, 55.6% of cases showed activating mutations in the RAS/RAF pathways. Variants in TP53 and KIT were detected at higher rates than in the general population. Veterans with prior history of melanoma were at significantly higher odds of having TP53 mutation (OR = 2.67, p = 0.04). This suggests that TP53 may be a marker for recurrent melanoma and possibly alternative exposures in the military population. This study provides new information regarding the genetics of melanoma in a Veteran population and early-stage melanomas, highlighting risk factors unique to this population and contributing to the conversation about preventing melanoma deaths in US Military personnel.

## Introduction

Melanoma incidence has increased significantly over the past three decades. It is currently the fifth most common cause of cancer in men and women in the United States, and in 2021, it accounted for an estimated 4,600 deaths in men and 2,580 in women (1, 2). These findings are especially concerning for the military population as studies have shown that military personnel are at increased risk for melanoma compared to the general population (3–5). With a higher proportion of Caucasian males, military personnel are often at increased sun exposure from operating at more equatorial latitudes compared to the general public, and lack of effective sun protection behaviors (5–9). Additionally, other non-ultraviolet (UV) exposures have been identified to contribute to melanoma risk, including industrial chemicals, polyvinyl chloride, ionizing radiation, and high altitude, especially dependent on the specific duties and occupational environment (10). For instance, while radiation and high altitudes have been studied to increase melanoma risk in airline pilots, air force pilots may be assumed to incur similar exposures.

The genetics and pathophysiology of melanoma have not been well-studied in the Veteran population. Establishing relationships between melanoma genetic mutations and military service and exposures is significant because it may create opportunities to improve prevention and screening as well as optimize treatment for Veterans.

In general, the relationship between UV exposure and the somatic genetic mutations in melanoma has yet to be completely elucidated, and the majority of research on melanoma has thus far been on advanced-stage tumors. Several pathways have been described, including germline mutations in *CDKN2A*, somatic mutations in *BRAF*, and *KIT* tyrosine kinase mutations. *BRAF*, a serine/threonine kinase in the MAPK signaling pathway, was first reported in 46-66% of melanomas (11, 12). *BRAF* mutations are believed to arise from UV damage, though they appear to be more common in skin intermittently exposed to the sun rather than chronically exposed and may also be more common in melanomas in younger patients, lending credence to the Intermittent Exposure Hypothesis (13–17). One thought is that intense intermittent sun exposure causes genetic damage while also triggering immunosuppression, while chronic exposure allows for photo-adaption (18). In contrast, melanomas from chronically sun-damaged skin or from sites not routinely exposed such as acral or mucosal sites do not typically carry *BRAF* mutations and would be more associated with *NRAS* and *KIT* mutations respectively (13, 19). Notably, *NRAS* mutations are associated with nodular subtypes of melanoma and with poorer outcomes (20). Given the equatorial locations of military deployment as well as the nature of military work, military personnel may be more likely to experience chronic occupational sun exposure. Intermittent sun exposure is more sporadic in nature and would be more characteristic of an office employee who only receives intense sun exposure on vacations, for example. Accordingly, melanomas in the military population would be less likely to originate from the *BRAF* pathway when compared to the general population, which was our hypothesis, though this has not been established prior to this study.

While in principal, understanding these distinct genetic pathways is critical in personalizing the different treatment options, such as vemurafenib for *BRAF*, imatinib for *KIT*, or binimetinib for *BRAF* and *NRAS*, this theory has not yet materialized in standard clinical practice aside from using BRAF and MEK inhibitor for BRAF mutation (21). Additionally, earlier stage tumors may provide clearer understanding of the initial drivers of malignant transformation. The purpose of this study is to characterize the genetic signature of early-stage melanomas from Veterans who were successfully screened and timely diagnosed, which may therefore shed light on pathogenesis of melanomas in this population and in turn influence clinical approach to prevention, screening, diagnosis, and treatment.

## Materials and Methods

### Study Population

Tissue samples of confirmed melanoma cases in a 7-year period, between January 1, 2010 to January 1, 2017 were obtained from the Iowa City VAMC. Inclusion criteria included age at least 18 years old, stage at diagnosis 0 to 2. Exclusion criteria included concurrent internal malignant disease, incomplete medical records, and unavailable or inadequate tissue sample. Demographic and clinical data such as gender, race, age, military branch, previous history of melanoma or non-melanoma skin cancer, family history of skin cancer, diagnosis date, tumor stage, primary tumor location, tumor subtype, histopathology, and treatment were obtained by chart review. All human studies were approved by the authors’ Institutional Review Board.

### Next-Generation Sequencing

Mutational analysis was performed using a custom AmpliSeq™ (Ion Torrent, Thermo Fisher Scientific, Waltham, MA) hotspot or targeted next-generation sequencing (NGS) panel of 25 genes having been reported mutated in melanoma including *BRAF*, *NRAS* and *TP53.* DNA was extracted from unstained sections from formalin-fixed paraffin embedded tissue blocks containing melanoma tumor cells, and 20ng of DNA was used for NGS library preparation. The libraries were bar-coded, clonally amplified, and sequenced on an Ion S5XL. The data were analyzed using the Torrent Suite Software followed by a laboratory-developed pipeline. The assay has an analytic sensitivity of 2.5% for single nucleotide variants (SNV) and small insertions and deletions. Adequate coverage was considered to be at least 250X, indeterminate coverage was considered to be 100-250X, and inadequate coverage was considered to be below 100X.

### Statistical Analysis

Firth-penalized logistic regression models were used to assess the association between patient and clinicopathologic characteristics on presence of *BRAF*, *NRAS*, and *TP53* mutations. Estimated effects of predictors are reported as odds ratios (OR) along with 95% confidence intervals. All statistical testing was two-sided and assessed for significance at the 5% level using SAS v9.4 (SAS Institute, Cary, NC).

## Results

Of 185 Veterans diagnosed with melanoma from January 1, 2010 to January 1, 2017 at the VAMC, there were 135 Veterans who met our cohort criteria. The demographics of this cohort is outlined in **Table 1** and shows a gender distribution of 96.3% male and 3.7% female. All 125 Veterans with reported race identified as Caucasian (100%) with 10 patients listed as having unknown race. Unknown values may be due to Veteran declining to answer or not being assessed for it. Mean age is 68.5 years. Military branch distribution was skewed towards the Army at 63.0% with 14.1% in the Navy, 11.9% in the Marines, and 11.1% in the Air Force.

**Table 1:** Demographics of cohort.

	Stage of Disease			
	0	1	2	All
	N (%)			
All	16	100	19	135
Gender				
Male	15 (93.8)	96 (96.0)	19 (100)	130 (96.3)
Female	1 (6.3)	4 (4.0)	0 (0)	5 (3.7)
Age Range				
18 – 29	1 (6.3)	2 (2.0)	0 (0)	3 (2.2)
30 – 49	1 (6.3)	10 (10.0)	1 (5.3)	12 (8.9)
50 – 64	1 (6.3)	21 (21.0)	3 (15.8)	25 (18.5)
65 – 79	8 (50)	53 (53.0)	12 (63.2)	73 (54.1)
80+	5 (31.3)	14 (14.0)	3 (15.8)	22 (16.3)
Mean Age (SD)	72.3 (16.1)	67.6 (13.2)	70.0 (11.8)	68.5 (13.3)
Race				
Caucasian	14 (100)	93 (100)	18 (100)	125 (100)
Unknown*	2	7	1	10
Ethnicity				
Hispanic	1 (6.7)	0 (0)	0 (0)	1 (0.8)
Non-Hispanic	14 (93.3)	97 (100)	19 (100)	130 (99.2)
Unknown*	1	3	0	4
VA branch				
Army	10 (62.5)	59 (59.0)	16 (84.2)	85 (63.0)
Navy	2 (12.5)	14 (14.0)	3 (15.8)	19 (14.1)
Marines	2 (12.5)	14 (14.0)	0 (0)	16 (11.9)
Air force	2 (12.5)	13 (13.0)	0 (0)	15 (11.1)
VA service yrs				
<2 years	2 (12.5)	6 (6.0)	2 (10.5)	10 (7.4)
2 years service	6 (37.5)	44 (44.0)	9 (47.4)	59 (43.7)
3 years service	3 (18.8)	18 (18.0)	5 (26.3)	26 (19.3)
4 years service	3 (18.8)	18 (18.0)	1 (5.3)	22 (16.3)
>4 years	2 (12.5)	14 (14.0)	2 (10.5)	18 (13.3)
Service-connected disability				
Yes	7 (43.8)	42 (42.0)	10 (52.6)	59 (43.7)
No	9 (56.2)	58 (58.0)	9 (47.4)	76 (56.3)
Service-connected disability for dermatologic condition				
Yes	2 (12.5)	7 (7.0)	2 (10.5)	11 (8.1)
No	14 (87.5)	93 (93.0)	17 (89.5)	124 (91.9)
Smoking status				
Former	5 (38.5)	47 (48.0)	5 (27.8)	57 (44.2)
Current	3 (23.1)	26 (26.5)	4 (22.2)	33 (25.6)
Never	5 (38.5)	25 (25.5)	9 (50.0)	39 (30.2)
Unknown*	3	2	1	6

VA, Veterans Affairs.

*As demographic data is obtained from medical records, there were some unknown values. This may represent either Veterans declining to answer demographic questions or never having been assessed for it.

Histopathologic features could be found in pathology reports of 110 Veterans of the 135 total Veterans included in this study. Twenty-five Veterans had pathology reports that did not include these features. Of the 110 samples, ulceration was noted for 7 (6.4%), mitoses were noted for 25 (22.7%), perineural invasion was noted for 2 (1.8%), regression was noted for 16 (14.5%), and desmoplasia was noted for 4 (3.6%). Tumor-infiltrating lymphocytes and microsatellitosis were assessed but not found in any of the 110 samples. For samples that were noted to have mitoses, the most common number of mitoses per mm^2^ noted was 1 (N=13), the greatest number of mitoses per mm^2^ noted in a single sample was 8, and the average number of mitoses per mm^2^ for the 25 samples that had them was 2.3. These results are summarized in **Table 2**. Immunohistochemistry was only performed on a small subset of these samples and as a result not evaluated. Univariate analysis of histopathologic features with regards to melanoma mutations did not show any with statistical significance.

**Table 2:** Histopathologic features in melanomas of 110 patients.

Feature	N	Percent
Ulceration	7	6.4
Mitoses	25	22.7
Perineural invasion	2	1.8
Regression	16	14.5
Tumor-infiltrating lymphocytes	0	0
Microsatellitosis	0	0
Desmoplasia	4	3.6

Histopathologic features could be found in pathology reports of 110 Veterans of the 135 total Veterans included in this study. Twenty-five Veterans had pathology reports that did not include these features. The table shows how many of each feature were noted and the percentage representation out of 110. In 25 samples with mitoses identified, the most common number of mitoses per mm^2^ noted was 1 (N=13), the max was 8, and the average was 2.3.

We analyzed each of the 135 cases of melanoma with next-generation sequencing targeting 25 hotspot mutations to profile the underlying genetic mutations in our cohort. Results of next generation sequencing is summarized in **Table 3** and shows 49 (36.3%) all-type *BRAF* mutations, 35 (25.9%) *TP53* mutations, 26 (19.3%) *NRAS* mutations, 15 (11.1%) *CDKN2A* mutations, 11 (8.1%) *KIT* mutations, and 10 (7.4%) *BAP1* mutations with the highest prevalence. Of the 49 *BRAF* mutations, 46.9% (23) were V600E mutations and 44.9% (22) were V600K. The remaining 8.2% (4) of BRAF mutations were V600N, S594N, N581I, and S607F. Of the 26 NRAS mutations, 38.5% (10) were Q61R mutations, 19.2% (5) were Q61K mutations, 19.2% (5) were Q61L mutations, 7.7% (2) were Q61H mutations, 3.8% (1) was a Q61P mutation, 11.5% (3) were G13R mutations, and 7.7% (2) were G12D mutations. Collectively, 84.6% (22) of NRAS mutations were in codon 61. Activating mutations in the RAS/RAF pathways, including *BRAF* V600E and V600K, *HRAS*, *NRAS*, and *RAF1* mutations, collectively comprised 75 (55.6%) of the cohort. Twenty-three patients (17.0%) had tumor biopsies that were negative for any of the gene mutations targeted, and 54 (40.0%) had biopsies positive for more than one mutation.

**Table 3:** Mutations found in melanomas of 135 patients by next-generation sequencing.

Gene	N	Percent
*AKT1*	1	0.7
***BAP1***	**10**	**7.4**
***BRAF***	**49**	**36.3**
***BRAF* V600E**	**23**	**17.0**
***BRAF* V600K**	**22**	**16.3**
***BRAF* V600N**	**1**	**0.7**
***BRAF* S594N**	**1**	**0.7**
***BRAF* N581I**	**1**	**0.7**
***BRAF* S607F**	**1**	**0.7**
***CDKN2A***	**15**	**11.1**
*CTNNB1*	1	0.7
*EIF1AX*	1	0.7
*ERBB4*	5	3.7
*FGFR1*	2	1.5
*FGFR2*	3	2.2
*FGFR3*	6	4.4
*GNA11*	2	1.5
*GNAQ*	2	1.5
*HRAS*	3	2.2
***KIT***	**11**	**8.1**
***KIT* L576P**	**3**	**2.2**
***KIT* D579N**	**2**	**0.7**
*MET*	4	3.0
***NRAS***	**26**	**19.3**
***NRAS* Codon 61**	**22**	**16.3**
***NRAS* G13R**	**3**	**2.2**
***NRAS* G12D**	**1**	**0.7**
*PDGFRA*	2	1.5
*PIK3CA*	3	2.2
*PTEN*	7	5.2
*RAF1*	1	0.7
*RB1*	3	2.2
*SF3B1*	2	1.5
*STK19*	4	3.0
***TP53***	**35**	**25.9**
***TP53* R282W**	**3**	**2.2**
***TP53* S241F**	**3**	**2.2**
***TP53* E286K**	**2**	**1.5**
***TP53* P278S**	**2**	**1.5**
*TRRAP*	0	0

Breakdown of specific mutations are shown for BRAF, NRAS, KIT, and TP53. Note that NRAS mutations involving codon 61, which typically codes for glutamine, had more variable substitutions and were thus grouped together. Likewise, KIT and TP53 had variable mutations and the full breakdown of mutations is not included in the table.

To understand the possible associations of demographic and clinical characteristics with the three most common mutations, we performed univariate analysis of the clinicopathologic data for *BRAF*, *NRAS*, and *TP53* mutations. Results for *BRAF* mutations are detailed in **Table 4**. Veterans who had melanoma primary tumor in the head/neck (OR = 0.30, 95% CI 0.12, 0.74) and the extremities (OR = 0.21, 95% CI 0.09, 0.52) were at decreased odds for *BRAF* mutation than those in the trunk (p <0.01) as is seen in other studies (16, 17). Increasing age was associated with decreased odds for having *BRAF* mutation (OR = 0.97, 95% CI 0.94–0.99, p = 0.01).

**Table 4:** Univariate analysis between demographic and clinical factors and *BRAF* mutations.

Variable	Odds of *BRAF* Mutation					
	Group	N	Odds Ratio	95% CI		P-value
Smoker	Former	57	1.03	0.43	2.48	0.13
	Current	33	2.33	0.89	6.10	
	Never	39	Ref	–	–	
VA Branch	Air Force	15	1.85	0.43	7.93	0.31
	Army	85	0.98	0.31	3.08	
	Navy	19	2.31	0.58	9.20	
	Marines	16	Ref	–	–	
Age at diagnosis	Units=1	135	0.97	0.94	0.99	**0.01**
Stage at diagnosis	1 or 2	119	1.23	0.41	3.74	0.71
	0	16	Ref	–	–	
Anatomic	Extremity	47	0.21	0.09	0.52	**<0.01**
	Head/Neck	39	0.30	0.12	0.74	
	Trunk	49	Ref	–	–	
Subtype	In situ	18	0.63	0.20	1.99	0.84
	Lentigo	39	1.09	0.48	2.49	
	Nodular	13	1.00	0.29	3.44	
	Superficial	59	Ref	–	–	
Personal history of melanoma	Yes	23	0.46	0.16	1.30	0.14
	No	112	Ref	–	–	
Personal history of NMSC	Yes	56	0.74	0.36	1.52	0.41
	No	79	Ref	–	–	
Family history of melanoma	Yes	8	1.81	0.43	7.60	0.42
	No	127	Ref	–	–	
Family history of NMSC	Yes	11	1.53	0.44	5.30	0.50
	No	127	Ref	–	–	

VA, Veterans Affairs; NMSC, Non-melanoma skin cancer.

Univariate analysis was performed on various clinical variables listed in the table to determine the odds of BRAF mutation. For each clinical variable, one group was assigned as a reference for which to compare the odds of other groups, hence the odds ratio of the chosen reference group is 1. Odds ratio for other groups listed within a clinical variable will be in comparison to the reference group odds of BRAF mutation.

**Figure 1:**
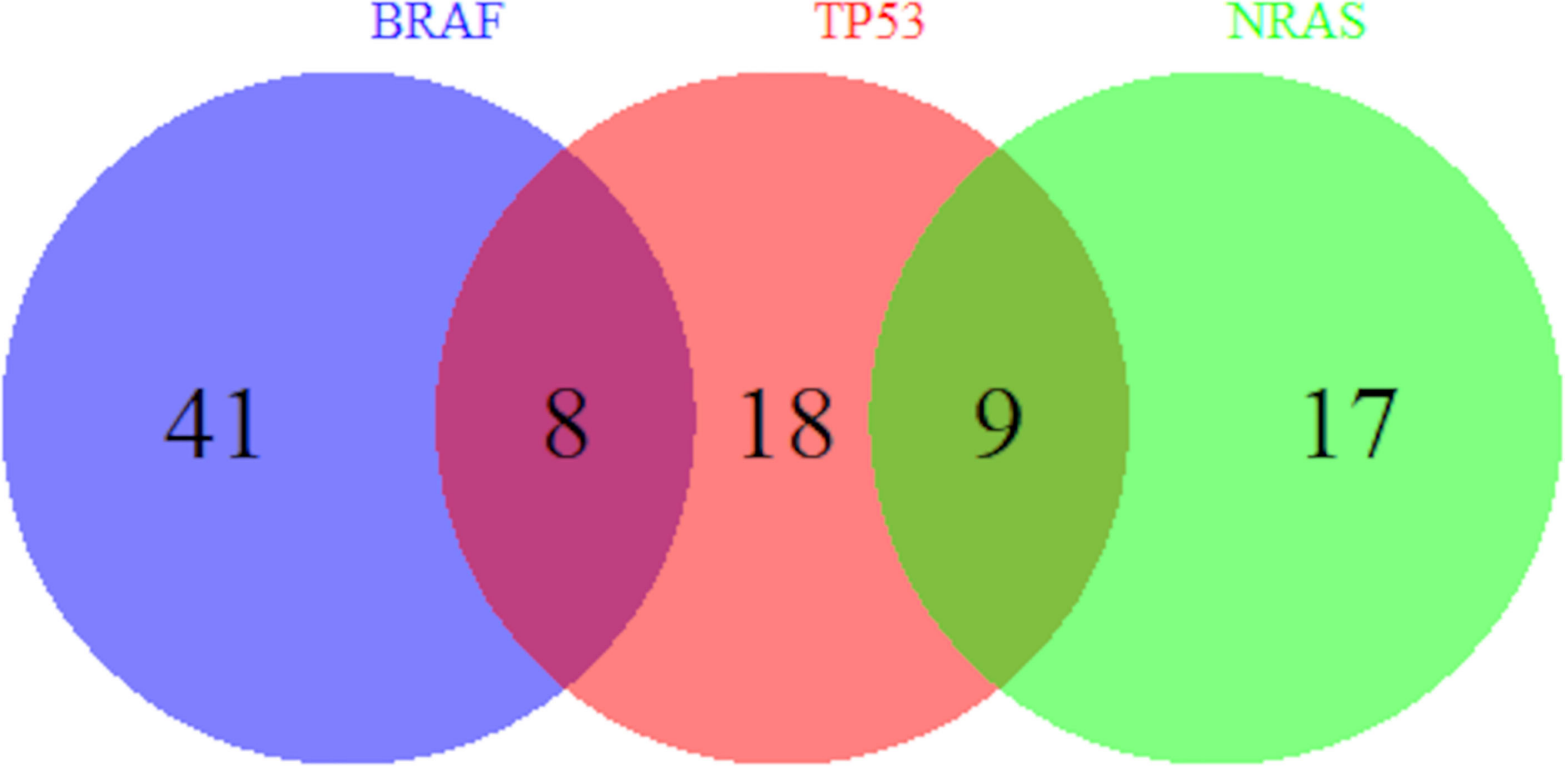
Overlap of *BRAF*, *TP53*, and *NRAS* Mutations. Number of Veterans with melanoma harboring *BRAF*, *TP53*, and *NRAS* mutations out of our cohort of 135 Veterans are shown. A subset of melanomas has both *BRAF* and *TP53* mutations and another subset has both *NRAS* and *TP53* mutations. However, *BRAF* and *NRAS* mutations are mutually exclusive.

Univariate analysis of clinicopathologic data with *NRAS* mutations are summarized in **Table 5**. Veterans who had melanoma primary tumor in the extremities were at increased odds of *NRAS* mutation than those in the trunk (OR = 2.03, 95% CI 0.79–5.20) while those with head/neck melanoma were at decreased odds (OR = 0.28, 95% CI 0.06–1.25) compared to those with trunk melanoma (p = 0.02) as seen in other studies (22–24). Compared to superficial spreading subtypes of melanoma, lesions that were lentigo maligna melanoma (OR = 0.16, 95% CI 0.04–0.67) were at decreased odds of having an *NRAS* mutation (p = 0.02). In addition, personal history of non-cutaneous cancer increased odds of *NRAS* (OR = 3.05, 95% CI 1.22–7.59, p = 0.02). Moreover, **Figure 1** shows which Veterans had melanomas with isolated or concurrent *BRAF*, *NRAS*, and *TP53* mutations. While some melanomas had either *BRAF* or *NRAS* with *TP53* mutations, *BRAF* and *NRAS* mutations were mutually exclusive.

**Table 5:** Univariate analysis between demographic and clinical factors and *NRAS* mutations.

Variable	Odds of *NRAS* Mutation					
	Group	N	Odds Ratio	95% CI		P-value
Smoker	Former	57	1.12	0.42	3.00	0.73
	Current	33	0.72	0.21	2.38	
	Never	39	Ref	–	–	
VA Branch	Air Force	15	1.62	0.26	10.30	0.73
	Army	85	1.70	0.39	7.42	
	Navy	19	0.83	0.12	5.76	
	Marines	16	Ref	–	–	
Age at diagnosis	Units=1	135	0.99	0.96	1.03	0.66
Stage at diagnosis	1 or 2	119	2.79	0.47	16.58	0.26
	0	16	Ref	–	–	
Anatomic	Extremity	47	2.03	0.79	5.20	**0.02**
	Head/Neck	39	0.28	0.06	1.25	
	Trunk	49	Ref	–	–	
Subtype	In situ	18	0.21	0.03	1.26	**0.02**
	Lentigo	39	0.16	0.04	0.67	
	Nodular	13	1.57	0.45	5.47	
	Superficial	59	Ref	–	–	
Personal history of melanoma	Yes	23	0.93	0.30	2.92	0.90
	No	112	Ref	–	–	
Personal history of NMSC	Yes	56	1.53	0.65	3.58	0.33
	No	79	Ref	–	–	
Family history of melanoma	Yes	8	1.62	0.33	8.12	0.55
	No	127	Ref	–	–	
Family history of NMSC	Yes	11	1.08	0.24	4.94	0.92
	No	124	Ref	–	–	

VA, Veterans Affairs NMSC, Non-melanoma skin cancer.

Univariate analysis was performed on various clinical variables listed in the table to determine the odds of NRAS mutation. For each clinical variable, one group was assigned as a reference for which to compare the odds of other groups, hence the odds ratio of the chosen reference group is 1. Odds ratio for other groups listed within a clinical variable will be in comparison to the reference group odds of NRAS mutation.

Results of univariate analysis of *TP53* mutations are summarized in **Table 6**. We observed that Veterans with prior history of melanoma were at increased odds of having a *TP53* mutation (OR = 2.67, 95% CI 1.05–6.80 p = 0.04). Unlike what we observed in our data for *BRAF* and *NRAS*, we did not find any association of *TP53* mutations with any anatomic location of melanoma or melanoma subtype. Unexpectedly, our results appear to indicate that neither smoking status nor military branch were associated with *BRAF*, *NRAS*, or *TP53* mutations.

**Table 6:** Univariate analysis between demographic and clinical factors and *TP53* mutations.

Variable	Odds of *TP53* Mutation					
	Group	N	Odds Ratio	CI 95%		P-value
Smoker	Former	57	0.83	0.33	2.07	0.91
	Current	33	0.96	0.34	2.69	
	Never	39	Ref	–	–	
VA Branch	Air Force	15	0.71	0.11	4.53	0.66
	Army	85	1.63	0.44	5.95	
	Navy	19	1.46	0.30	7.07	
	Marines	16	Ref	–	–	
Age at diagnosis	Units=1	135	1.00	0.97	1.03	0.97
Stage at diagnosis	1 or 2	119	0.53	0.18	1.57	0.25
	0	16	Ref	–	–	
Anatomic	Extremity	47	1.19	0.45	3.09	0.25
	Head/Neck	39	2.14	0.83	5.52	
	Trunk	49	Ref	–	–	
Subtype	In situ	18	2.25	0.73	6.92	0.55
	Lentigo	39	1.07	0.41	2.80	
	Nodular	13	1.15	0.28	4.64	
	Superficial	59	Ref	–	–	
Personal history of melanoma	Yes	23	2.67	1.05	6.80	**0.04**
	No	112	Ref	–	–	
Personal history of NMSC	Yes	56	1.47	0.68	3.19	0.33
	No	79	Ref	–	–	
Family history of melanoma	Yes	8	0.54	0.08	3.63	0.53
	No	127	Ref	–	–	
Family history of NMSC	Yes	11	0.37	0.06	2.34	0.29
	No	127	Ref	–	–	

VA, Veterans Affairs; NMSC, Non-melanoma skin cancer.

Univariate analysis was performed on various clinical variables listed in the table to determine the odds of TP53 mutation. For each clinical variable, one group was assigned as a reference for which to compare the odds of other groups, hence the odds ratio of the chosen reference group is 1. Odds ratio for other groups listed within a clinical variable will be in comparison to the reference group odds of TP53 mutation.

## Discussion

The pathogenesis of melanoma development, including relationships to genetic mutations, continues to be elucidated. However, there is currently a dearth of research on melanoma in military personnel. In this study, we have been able to profile tumor hotspot mutations in early-stage melanomas in a veteran population.

Evaluation of the 110 samples with reported histopathologic features in patient medical records show that relatively few of the samples had these notable features. These features, including ulceration, mitoses, and perineural invasion, generally suggest more invasive tumors and have been correlated with poorer prognosis. It is unclear why twenty-five samples did not include evaluation of these features, which may limit interpretation. However, the data is consistent with having lower-staged melanomas, which were purposefully selected for this study with intention of identifying earlier features and mutations in pathogenesis. Univariate analysis was performed on histopathologic features and odds of melanoma mutations, but statistical significance was not found. Given the low feature count, we believe that this analysis lacked statistical power to identify significance if any were present.

Of 135 Veterans whose melanoma was analyzed by next-generation sequencing, 49 (36.3%) had *BRAF* mutations, 26 (19.3%) had *NRAS* mutations, and 35 (25.9%) had *TP53* mutations, which were the three most common mutations. *BRAF* was initially found to be in 44-66% of melanomas in the general population, and that has since been corroborated with other reports in that range (11, 12). Our cohort had a lower prevalence of *BRAF* compared to what has been reported in the general population, which supports the idea that Veterans incur chronic sun exposure rather than intermittent sun exposure, though our population was also older. This is in line with the equatorial locations that many Veterans are frequently stationed at globally and domestically as well as previous work that has reported sun protection education and practice gaps in the military (5–9). Moreover, nearly half (44.9%) of the *BRAF* mutations were V600K mutations, which is greater than the 10–30% that has been reported in other studies (25, 26). *BRAF* V600K mutations have been more associated with chronic sun exposure compared to V600E mutations as well as older age and higher risk of metastasis, which suggests a different pathology than the more common V600E mutation (27, 28). In our cohort, *BRAF* was associated with younger age and tumor location in the trunk in this cohort, which are similar findings to what have been reported in the general population (16, 17, 29, 30). While the mean age of diagnosis of melanoma in the general population is 63, the mean age of our cohort approaches 69 years old, which corroborates the lower *BRAF* prevalence and greater percentage of V600K mutations (31).

*NRAS* mutation prevalence in this cohort was found to be within a comparable range of what has been reported for the general population (19% vs. 20%) (22). This is surprising considering that NRAS is associated with chronic sun damage and would therefore be expected to be at higher prevalence in this population given chronic occupational sun exposure, though that is not the case here. One possibility is that the pathogenesis involving greater cumulative sun exposure in this veteran population favors *BRAF* V600K over *NRAS* mutations. As shown in other studies, *NRAS* and *BRAF* mutations were mutually exclusive, showing distinct pathogenesis (11, 30, 32). Our study showed NRAS to be more common on the extremities, which again supports the connection with chronically sun-damaged skin. Nodular melanoma subtype and extremity anatomic location were also found to be at higher odds for *NRAS*, which agrees with what has been reported in the general population (22–24). Moreover, the breakdown of mutations in *NRAS*, with general predominance of codon 61 mutations, and more specifically Q61R, has been noted in other studies (33, 34). This may suggest that Veterans undergo similar pathogenesis as the general population in *NRAS* mutations, though exact statistical comparison is challenging given the low number of mutations observed. These findings raise the question of how much the genetic profile described in this veteran population results from chronic sun exposure as opposed to other risk factors that have so far not been well-examined, including chemical exposures and ionizing radiation.

*TP53* mutation prevalence in this cohort was also higher than what has been reported for the general population (26% vs. 15-20%) (35, 36). Interestingly, having a previous history of melanoma before the current diagnosis for this study was associated with increased odds of having *TP53* mutations. This suggests that *TP53* mutations may be associated with increased risk for recurrence of melanoma. In one study, wild-type p53 enzyme was correlated with a longer relapse-free period in melanoma patients (37). That would suggest that p53 plays an important suppressive role in preventing melanoma tumorigenesis, and that *TP53* mutations may disinhibit melanoma development, leading to recurrence of melanoma. Understanding *TP53* subtypes could therefore be key to stratifying risk in the general population or even specifically within the military, which is especially critical given its higher prevalence in this population.

It is worth noting that *KIT* mutations, which are generally uncommon and have a reported 1-5% prevalence, was found to be 8% in our cohort (38, 39). *KIT* mutations are typically found in melanomas on mucosal and acral areas, which points toward non-UV exposures. Higher *KIT* mutations in Veterans therefore further suggests greater significance of other non-UV related risk factors in the military population. These may also explain melanomas in more varied Fitzpatrick skin types, though our study was limited by access to only types I-III. Unfortunately, the sample size of *KIT* mutations was not large enough to draw statistically meaningful relationships to demographic and clinical data.

These findings help shed light on melanoma in the Veteran population. Few studies have been conducted on trying to understand the pathogenesis of melanoma in military populations, and none have investigated the genetic profile of their melanomas. Additionally, because the study was conducted on earlier staged disease, it is more likely to show the initial drivers of carcinogenesis rather than cumulative mutations over time. This will help better elucidate the pathways that lead to melanoma development in this population. In combination with demographic information and clinic history, associations to exposures and risks can made. For instance, stronger relationships to cumulative sun exposure as well as non-UV exposures have been hinted by the lower prevalence of *BRAF* and increased *KIT* compared to what studies have found in the general population.

However, more work needs to be done to fully understand the exact exposures and mechanism of pathogenesis. This study primarily covered hotspots known to be commonly found in tumors, but other mutations may be missed. Additionally, the cohort consisted of only patients seen at the Iowa City VAMC and with a skew towards Army branch, which may not be completely representative of the military or Veteran population as a whole. Validation at other sites may be important in this regard. Lastly, this study did not have a matched control for direct comparison. While many studies have outlined the general rates and prevalence of mutations in melanoma in the general population, having a matched control would improve validity and increase the sensitivity of detecting significant deviations from a controlled sample.

In conclusion, we were able to profile 25 hotspot gene mutations in early-stage melanoma in Veterans, which showed lower prevalence of *BRAF*, higher *KIT* and *TP53*, and comparable *NRAS* mutations compared to what has been reported for the general population. In doing so, we were able to shed light on the unique genetic signatures that may be seen in this population. The lower prevalence of *BRAF* mutations and higher percentage being *BRAF* V600K points toward cumulative sun damage as a larger risk factor for Veterans, which in combination with previous studies showing poor sun protection education and practices in the military, strongly advocates for improvement in this regard. The higher *KIT* prevalence suggests increased non-UV risk factors, which will need to be further explored to identify and understand these exposures in order to improve prevention practices. *TP53* mutations was more likely in individuals with previous history of melanoma, which identifies a subpopulation of Veterans who may need closer evaluation of melanoma recurrence. While this study provides new information regarding both genetics of melanoma in a Veteran population and early-stage tumors, more work will need to be done in order to better understand the exact role that these mutations play in pathogenesis. Future studies may include comparative studies with matched controls, validation at other VA medical centers, larger studies to increase statistical power, and expansion of the gene mutation panel to identify other drivers of malignancy. A follow-up study for this cohort may also be considered, though it is unclear how many of these Veterans will continue to receive routine care at the Iowa City VAMC. Ultimately, these findings should influence how we educate, screen, and treat melanoma in Veterans and active military personnel, and pave the way for continued research in this higher risk population.

## Data Availability Statement

The datasets presented in this study can be found in online repositories. The names of the repository/repositories and accession number(s) can be found below: http://dx.doi.org/10.17632/sk7g7wncms.1.

## Ethics Statement

The studies involving human participants were reviewed and approved by University of Iowa, Human Subjects Office/IRB. Written informed consent for participation was not required for this study in accordance with the national legislation and the institutional requirements.

## Author Contributions

JP conceived the original idea, acquired funding, supervised the research study, and helped design the methodology. KC wrote the original draft, helped design the methodology, and assisted in investigation and data curation. AB assisted in investigation, provided resources and methodology for genetic analysis, and helped review and edit the manuscript. SM led statistical analysis, supported data curation and methodology, and reviewed the manuscript. MZ supported statistical analysis and data curation and reviewed the manuscript. JM assisted with project administration and data curation. YZ helped review and edit the manuscript. BS provided access to Veteran Affairs samples and helped review and edit the manuscript. All authors have reviewed the author contributions and agree that the role designations are correct.

## Funding

This research was funded and supported by the University of Iowa Department of Dermatology.

## Conflict of Interest

The authors declare that the research was conducted in the absence of any commercial or financial relationships that could be construed as a potential conflict of interest.

## Publisher’s Note

All claims expressed in this article are solely those of the authors and do not necessarily represent those of their affiliated organizations, or those of the publisher, the editors and the reviewers. Any product that may be evaluated in this article, or claim that may be made by its manufacturer, is not guaranteed or endorsed by the publisher.
